# Evaluating client functioning in a harm reduction program in South Africa: insights from a tool derived from the International Classification of Functioning, Disability and Health

**DOI:** 10.3389/fresc.2024.1445176

**Published:** 2024-12-19

**Authors:** Michelle N. S. Janse van Rensburg, Daleen Casteleijn, Andrew Scheibe

**Affiliations:** ^1^ Community Oriented Primary Care (COPC) Research Unit, Faculty of Health Sciences, University of Pretoria, Tshwane, South Africa; ^2^ Department of Occupational Therapy, Faculty of Health Sciences, University of the Witwatersrand, Johannesburg, South Africa; ^3^ Department of Occupational Therapy, Faculty of Health Sciences, University of Pretoria, Tshwane, South Africa

**Keywords:** substance use, functioning, harm reduction, ICF, people who use drugs, COSUP, South Africa

## Abstract

South Africa faces the detrimental effects of problematic substance use. The Community Oriented Substance Use Program (COSUP) is a research-based, community-situated harm-reduction program. The International Classification of Functioning, Disability and Health (ICF) was used as the framework to develop a unique tool to determine the functioning of COSUP clients. The study was a quantitative descriptive, cross-sectional design, with data collected from COSUP sites during January 2023 using the COSUP Client Functioning Tool. Twenty-three Likert-scale structured closed questions about clients’ perceptions of their functioning and context were analyzed using descriptive statistics. Open-ended questions about COSUP services were thematically analyzed. Most COSUP clients are working-age African males, and many are unemployed. Clients seem to be coping well physically but need more mental health support. Pressing concerns for COSUP clients are feeling stressed and anxious, an inability to handle stress, poor use of free time, not getting support from others, and not having enough money to meet daily needs. Lack of energy and boredom are significant concerns, along with feelings of rejection and loneliness. Facilitating opportunities for sustaining livelihoods requires focus. Even so, there are those who have a sense of hope due to the positive impact of the program. Basing the COSUP Client Functioning Tool on the ICF framework provided a useful picture of the functioning of people who use/d drugs in their contexts. The COSUP Tool is helpful to guide interventions that are responsive to clients’ needs.

## 1 Introduction

The global landscape of substance use disorders presents a multifaceted challenge, with implications for individual health and for societal well-being (1). South Africa, as many other countries, faces the detrimental effects of problematic substance use, with opioids, particularly heroin (locally called *nyaope)* emerging as a significant concern (2). The South African Community Epidemiology Network on Drug Use's biannual reports underscore the gravity of the situation, indicating high rates of opioid use and associated harms, especially among vulnerable populations (3).

In 2020 in South Africa there were an estimated 400 000 people who used heroin, 350 000 people who use cocaine and 290 000 people who use methamphetamine, with an estimated 82 500 people who inject drugs (4). HIV and hepatitis C prevalence among people who inject drugs in 2017 was estimated at 21%–53%, respectively (5). Data on mortality among people who use drugs is limited, with a small pilot study (2019) suggesting that drug-related overdose is occurring; with two-thirds of people knowing someone who had experienced an overdose (6). A recent review of medical insurance databases identified that people with opioid-related disorders had a 7.8-year shorter life expectancy than those people without (7).

The Community Oriented Substance Use Program (COSUP) is a collaboration between the City of Tshwane Metropolitan Municipality (CoTMM) and the University of Pretoria's Community Oriented Primary Care (COPC) Research Unit, and is the only publicly funded, community-based harm reduction program responding to the use of illegal substances in the country (8). COSUP was established in 2016 in Tshwane due to the recognition of substance use as a complex public health and social issue requiring holistic and community-driven interventions (8), and currently operates across 17 community sites in Tshwane. The limitations of “traditional” abstinence-based treatment modalities, such as inpatient rehabilitation, along with the dearth of public-sector rehabilitation services in South Africa, leaves many individuals without access to timely and appropriate care, and highlights the need for other approaches that are responsive to people's lived realities (9).

COSUP provides pragmatic strategies, such as a needle and syringe program, opioid agonist maintenance therapy (OAMT), counselling, group therapy and opportunities for skills development, aimed at minimizing the negative consequences of substance use without requiring cessation. Services are provided through interdisciplinary teams, which include doctors, clinical associates, social workers, peer educators, community health workers, and information officers (8), with some sites also having psychology and occupational therapy services through student work-integrated learning placements.

As the program progressed, there was a need for information regarding the quality of life of COSUP clients. While quality of life measures exist, they are not necessarily contextually relevant or specific to people who use drugs. The World Health Organization's International Classification of Functioning, Disability and Health (ICF) (10) was selected as the preferred framework to guide the development of a tool to assess and monitor the functioning of COSUP clients to align services with clients’ needs.

The ICF considers the whole person existing within a complex context. It is useful to determine clients’ improvements in functioning over time (10). Functioning is an umbrella term for different components such as body functions and body structures, and activities and participation, along with consideration of an individual's context, i.e., environmental factors and personal factors. This provides an integrated perspective of biological health (the capacity for health in terms of body functions and structures) and lived health (participation in life activities within a person's context) (11). Thus, apart from the biomedical or psychosocial considerations of substance use, the ICF, as a person-centered framework, facilitates a bio-psycho-social-spiritual perspective of clients functioning within their contexts (12, 13).

The COSUP Functioning Tool (hereafter referred to as the COSUP Tool) was locally developed by COSUP team members who identified the need for an assessment tool specific to individuals using substances in a harm reduction program and in a low socio-economic environment. In recent years, the use of Patient-Reported Outcome Measures (PROMs) and Patient-Reported Experience Measures (PREMs) have become an international standard in healthcare for assessing treatment outcomes and quality of care from the patient's perspective (14). These measures allow for individualized feedback and highlight patient/client experiences, offering valuable insights for service improvement in substance use disorder (SUD) treatment (15).

Our research builds on this international trend by focusing on the assessment of functioning as a crucial dimension of client outcomes in substance use intervention, which complements PROMs and PREMs. While PROMs provide insights into perceived care quality and self-reported treatment outcomes, our functioning tool addresses specific challenges and needs unique to individuals using drugs, enabling a holistic evaluation of intervention impact. PREMs focus on various aspects of care, including practical concerns such as accessibility and communication with service providers, and our tool includes questions about whether clients receive the services they need, the extent of emotional support provided, and their perceptions of COSUP's contribution to their lives.

The ICF focuses on understanding health and disability in the context of an individual's overall functioning within their unique environment. This client-centered perspective emphasizes the individual's lived experience, considering their abilities, challenges, personal goals, and social contexts. In doing so, it aligns with PROMs and PREMs, which prioritize the client's own perspective and experience of their health outcomes and quality of life.

The COSUP Tool was validated using the Rasch Measurement Model (RMM) through an iterative process of four rounds of data collection between 2019 and 2022. The first version of the questionnaire contained 18 Likert scale questions (five options to choose from) and was implemented in 2019. One hundred and twenty-eight completed questionnaires were returned to establish the face validity of the tool. COSUP social workers reviewed the responses and made changes based on face value (e.g., questions were rephrased or split for clarity). The second round contained 24 Likert scale questions and one open-ended question and was implemented in 2020 with 266 completed questionnaires returned. These questionnaires were subjected to Rasch analysis to determine the internal construct validity of the tool. The results showed that the Likert scale questions with five options were confusing for COSUP clients. After collapsing adjacent options in the scale, the Rasch analysis showed some questions worked well with only two options (e.g., *Do you experience physical pain?* and *Do you struggle with sleep?*). Other questions worked better with three options (e.g., *Do you have opportunities to acquire new skills?*), while most questions were clear with four options (e.g., carry out daily activities, feel bored, support from others). A few questions worked with five options e.g., *How happy are you with your (quality of) life, Are you worried or anxious*, *Do you experience loneliness or rejection*? A few questions showed redundance (already covered by other questions) e.g., opportunity for leisure activity and opportunity for community participation. These two questions were removed, after which the Rasch analysis revealed that the questions target the needs of the clients well and that no questions were confusing.

A third round was conducted in 2021, and 401 questionnaires were returned. The changes in the previous round paid off and the 22 questions and Likert scale options worked well. The open-ended question remained the same. The tool was ready to be rolled out for routine use. A fourth round was completed in the beginning of 2022 with 301 questionnaires returned. Another round of Rasch analysis was done and Rounds 3 and 4 data were combined (total sample of 702). The strengths of the questionnaire were confirmed e.g., the questions targeted the COSUP clients well, the measurement of their functioning was accurate and all questions with the varying options were clear.

A manuscript describing the development and psychometric properties of the COSUP tool is under review for publication at the time of writing this report.

This brief report highlights the application of the ICF framework in assessing and understanding the holistic functioning of individuals in a harm reduction program within the context of substance use during the most recent period of data collection, conducted at the end of 2022, using the COSUP Tool.

## 2 Method

The study employed a quantitative descriptive, cross-sectional design to present the holistic functioning of individuals using substances in a harm reduction program in South Africa. Data were collected with the fifth version of the COSUP Tool. Structured self-report questions captured demographic characteristics (age, gender, race, marital status, education and employment). A question on how COSUP clients utilized services (OAMT, counselling, psychosocial group interventions and skills development) was noted. Twenty-two Likert-scale structured closed-ended questions, containing two to five response categories, were used to assess clients’ perceptions about their functioning and context (including satisfaction with life, physical health, aspects of daily living, interpersonal relationships, safety and security, and sustaining livelihoods). One open-ended question, namely, *How do you feel about COSUP's contribution in your life?*, was included.

Social workers across all 17 COSUP sites received training on using the COSUP Tool, with a focus on the ICF framework that underpinned the questionnaire design. Paper-based copies of the Tool were sent to all COSUP sites, and clients accessing OAMT and/or psychosocial services were asked to complete it as a self-report. In cases where assistance was needed, social workers supported clients in filling out the questionnaire.

During the data collection period from October to December 2022, all COSUP clients who visited any of the 17 sites were asked to complete a questionnaire, irrespective of whether it was their first time attending or if they were returning clients. One of the demographic questions in the survey specifically asked participants if they had previously completed the questionnaire.

Fourteen of the 17 sites submitted completed questionnaires (three sites did not send back their questionnaires in time), yielding 450 responses. Completed questionnaires were collected and data were uploaded to Qualtrics (Provo, UT) by COSUP information officers.

**Figure 1:**
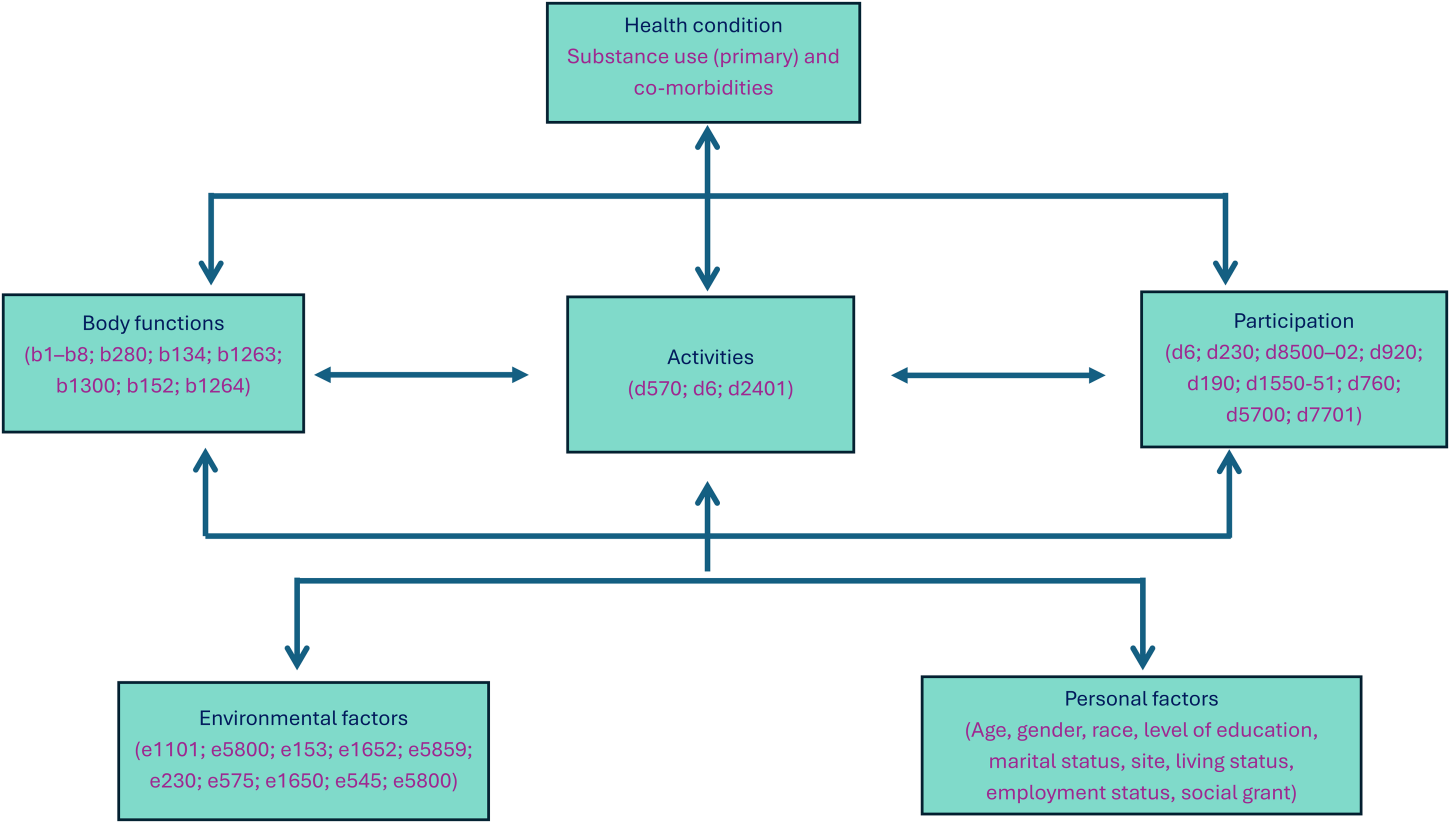
Distribution of ICF codes among the components of body functions, activities and participation, and environmental factors. LoE, level of education.

Data analysis was done by identifying the ICF codes for the questions, using the search function in the ICF browser (16) and then grouped according to the ICF components (Figure 1).

Descriptive statistics such as means and medians (numerical variables), proportions and frequencies (categorical variables) were analyzed using Microsoft Excel version 2405. Since the scale of the COSUP Tool is an ordinal scale, medians for each question were calculated and then reworked to a percentage for easy interpretation. For ease of reporting, the five category scales were collapsed into Yes/Neutral/No during data analysis, while ensuring no loss of information. The four category scales were reported in their original form.

The open-ended question provided an opportunity to give reasons for COSUP's contribution to clients’ lives and 277 responses were obtained. These were written mostly as phrases or sentences, which were analyzed using content analysis (17).

This research was done as part of COSUP's annually renewed ethical approval (University of Pretoria's Faculty of Health Sciences Research Ethics Committee, reference no. 83/2017 and 310/2020).

## 3 Results

Completed questionnaires were obtained from 14 sites. Data from 450 completed questionnaires were analyzed, however, there were missing data at different questions (data field not completed by the client or social worker), hence the variations in sample size for the subsections analyzed.

Of the respondents, 176 indicated they had completed it before, 219 said they had not, and 54 did not respond to this question. Additionally, 223 respondents indicated when they had started accessing COSUP services, of which 88 had been part of the program for 1–3 years and 37 for more than 3 years. Eighty-six respondents had joined the program within the past year, with 61 only joining in the past three months. This approach allowed us to capture data from both new and returning clients, ensuring a comprehensive representation of the population accessing COSUP services during the survey period.

Among the respondents, 234 reported receiving OAMT, 117 did not, and 99 left this question unanswered. COSUP's previous records indicate that 887 individuals accessed OAMT services between January and June 2022, though this figure excludes those utilizing other services such as counselling or skills development. In terms of the different types of services utilized by the clients at the combined locations, psychosocial group interventions had the highest attendance at 56% (*n* = 252) followed by OAMT at 52% (*n* = 234). Counselling services were utilized by 50% of participants (*n* = 226), while accessing skills training was reported by 18% (*n* = 81). In our results, of the respondents receiving OAMT, 202 service users also accessed counselling or group therapy services, half of whom accessed both on a regular basis (i.e., weekly or at least monthly).

Although the exact response rate could not be determined, we believe this sample size represents a substantial portion of the population attending COSUP sites and we consider the response rate to be robust. This suggests the survey successfully captured insights from a significant portion of the community, providing valuable data to guide program effectiveness and outreach strategies.

Table 1 outlines demographic data. Most clients were single, African males in the working-age range, with 20–29 and 30–39 years being the dominant age groups. The demographic information for level of education had almost 40% of missing data, thus, the result 85.9% of clients of 269 clients having a high school education should be interpreted with caution as it might not accurately reflect the educational background of this population. Unemployment is a concern for most of the clients, although some clients report being able to find occasional informal work.

**Table 1:** Personal factors as represented through demographic data (January 2023).

Age group (*n* = 427)	Male *n* = 385 (%)	Female *n* = 42 (%)	Race *n* = 442 (%)		Marital status *n* = 444 (%)		Level of education *n* = 269 (%)		Employment status *n* = 411 (%)	
16–19 years	9 (2.1)	1 (0.2)	African	357 (80.8)	Single	366 (82.4)	Primary school	8 (3.0)	Employed	47 (11.4)
20–29 years	86 (20.1)	7 (1.6)	Colored	36 (8.1)	Married	23 (5.2)	High school	231 (85.9)	Own business	13 (3.2)
30–39 years	211 (49.4)	21 (4.9)	Indian	11 (2.5)	Living together	32 (7.2)	Tertiary	24 (8.9)	Informal work	46 (11.2)
40–49 years	67 (15.7)	9 (2.1)	White	38 (8.6)	Separated	9 (2.0)	Post Grad	4 (1.5)	Piece jobs when available	122 (29.7)
50–62 years	12 (2.8)	4 (0.9)			Divorced	8 (1.8)	None	2 (0.7)	Unemployed, looking	152 (37.0)
Mean age	**34.27**	**36.4**			Widowed	6 (1.4)			Unemployed, not looking	31(7.5)

Bold values indicate the mean age.

Table 2 shows the descriptive analysis of the questions in the COSUP Tool around functioning (body functions, and activities and participation) and environmental factors, with the aligning ICF codes. In terms body functions, most clients did not have other chronic physical (77.7%, *n* = 335) or mental health (88.3%, *n* = 379) conditions, and did not experience physical pain (79%, *n* = 338) or difficulty sleeping (60.6%, *n* = 260). Just over half (52.5%, *n* = 219) of the participants felt that they had enough energy for the day, while 68.6% (*n* = 282) often felt bored. Even so, 72.6% (*n* = 291) of participants felt hopeful about their future.

**Table 2:** Results of functioning and environmental factors and corresponding ICF codes (COSUP participants, *n* = 450) (January 2,023).

Body functions				
COSUP tool question and ICF codes		Scale/scoring categories		
		Yes (%)		No (%)
Chronic physical condition/disability [b2-b8] (*n* = 431)		96 (22.3)		335 (77.7)
Chronic mental health condition/disability [b1; d570; e1101] (*n* = 429)		50 (11.7)		379 (88.3)
Sleep difficulty [b134] (*n* = 429)		169 (39.4)		260 (60.6)
Physical pain [b280] (*n* = 428)		90 (21)		338 (79)
Enough energy for everyday life [b1300] (*n* = 417)		219 (52.5)		198 (47.5)
Often feeling bored [b1264; d920] (*n* = 411)		282 (68.6)		129 (31.4)
Hopeful about the future [b1265] (*n* = 401)		291 (72.6)		110 (27.4)
		Yes (%)	Neutral (%)	No (%)
Happy with life [b1263; d6] (*n* = 413)		112 (27.1)	107 (25.9)	194 (47)
		Seldom (%)	Neutral (%)	Often (%)
Feeling worried, stressed or anxious [b152] (*n* = 386)		92 (23.8)	135 (35)	159 (41.2)
Feeling rejection or loneliness [b152] (*n* = 405)		180 (44.4)	94 (23.2)	131 (32.4)
Feeling negative, sad or depressed [b1263; b152] (*n* = 402)		145 (36.1)	137 (34)	120 (29.9)
Activities and participation				
	Very/always positive (very good)	Mostly positive (good)	Somewhat positive (moderate)	Mostly negative (poor)
Satisfied about health [d570] (*n* = 405)	104 (25.7)	147 (36.3)	92 (22.7)	62 (15.3)
Carry out daily activities [d230] (*n* = 410)	172 (41.9)	120 (29.3)	66 (16.1)	52 (12.7)
Handle stress [d2401] (*n* = 407)	51 (12.5)	122 (30)	159 (39.1)	75 (18.4)
Use free time constructively [d190; d920] (*n* = 403)	71 (17.6)	125 (31)	129 (32)	78 (19.4)
Satisfied with personal relationships [d7500; d7701] (*n* = 395)	96 (24.3)	161 (40.8)	85 (21.5)	53 (13.4)
Rate ability to work [d8500–8502; e153; e1652; e5859] (*n* = 413)	146 (35.4)	80 (19.4)	153 (37)	34 (8.2)
Environmental factors				
	Always	Mostly	Sometimes	Not at all
Satisfied with family relationships [e153; e1652] (*n* = 403)	106 (26.3)	135 (33.5)	96 (23.8)	66 (16.4)
Getting support from others [e230; e575] (*n* = 401)	74 (18.4)	93 (23.2)	151 (37.7)	83 (20.7)
Enough money to meet your need [e1650] (*n* = 404)	26 (6.4)	39 (9.7)	164 (40.6)	175 (43.3)
Feeling safe in your daily life [e545] (*n* = 406)	83 (20.4)	143 (35.2)	105 (25.9)	75 (18.5)
Positive about COSUP contribution [e5800] (*n* = 389)	247 (63.5)	101 (26)	27 (6.9)	14 (3.6)
	Yes		No	
Access to chronic medication [e1101] (*n* = 290)	142 (49)		148 (51)	

Regarding activities and participation, almost half of participants (47%, *n* = 194) were not happy with life. Overall, 32% (*n* = 131) reported to often have feelings of rejection or experiencing loneliness.

Environmental barriers include that half of the COSUP clients who needed chronic medication reported challenges accessing it (51%, *n* = 148), clients did not always feel safe in daily life (79.6%, *n* = 323), and, most especially, clients were not able to always meet their needs financially (93.6%, *n* = 378). Most participants reported always feeling positive about COSUP's contribution to their lives (63.5%, *n* = 247).

**Figure 2:**
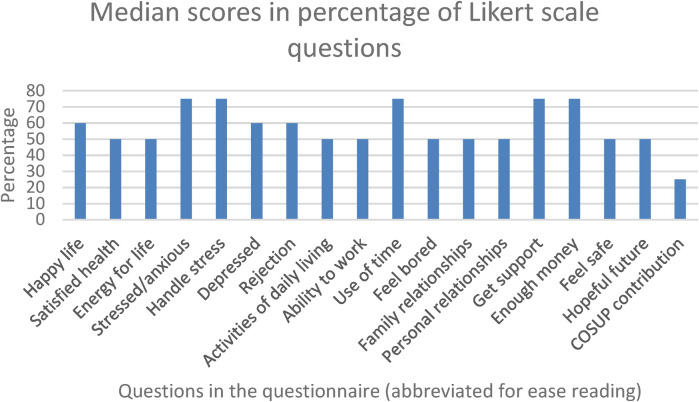
Average % of median scores per question (the higher the percentage, the poorer the functioning).

The results of the level of functioning in the COSUP clients are displayed in Figure 2 (Note: the higher the percentage on the graph, the poorer the functioning). The most pressing concerns for COSUP clients, at 75%, are feeling stressed and anxious, an inability to handle stress, poor use of free time, not getting support from others, and not having enough money to meet their daily needs. There were no aspects of functioning that were below 50%, except for the COSUP contribution, which indicates the positive contribution clients feel that COSUP makes in their lives.

From the 277 responses to the open-ended question, some of the positive findings revealed that COSUP has helped service users to reduce or stop the use of drugs, and even saved lives. Some clients reported that they had experienced a general improvement in their lives, especially in activities of daily living, such as selfcare and hygiene. Being supported through acceptance and non-judgement by COSUP staff, along with access to OAMT, counselling and support groups helped COSUP clients cope and feel a sense of gratitude for second chances. Improvements in relationships with family and the community provided hope. For some, the benefit of no longer being homeless was important. For several respondents these positive changes resulted in their being able to achieve their goals, as well as look for work or find work.

## 4 Discussion

The ICF framework was successfully applied to assess and comprehend individuals’ holistic functioning within a community-orientated substance use harm reduction program in a low-to-middle-income country (LMIC). In alignment with PROMs and PREMs, the COSUP Tool emphasizes personal experience by enabling clients to report on their perceived functioning, symptoms, and quality of life. This alignment with PROMs and PREMs makes the COSUP Tool essential in client-centered care, as it underscores the importance of evaluating outcomes that hold meaningful relevance to clients, reinforcing a holistic view of health and well-being.

In terms of the physical body functions, clients generally appeared to be coping well, which is likely due to the benefits of the biomedical services (such as OAMT) received through COSUP. However, the mental health-related body functions, such as feelings of worry, stress and anxiety, are pertinent. While counselling and group therapy services are well attended, the need for support among COSUP clients clearly is great, which reinforces the necessity of additional psychosocial services. The results of this tool can be used to inform tailored psychosocial management plans for people and be adjusted over time in response to changes identified through repeated tool administration.

Where clients coped the best was their ability to carry out daily activities, which is encouraging and indicative that COSUP services may mitigate chaotic substance use, as people who use drugs in this manner are unlikely to be able to conduct their important activities of daily living (8).

The high level of satisfaction reported by many clients regarding their family relationships may speak to the fruits of the efforts of COSUP social workers, community health workers (CHWs) and peer educators in working with families and households of clients in the program. Given that family relationships with PWUD are often strained (18), the observed satisfaction suggests that social workers and CHWs are successfully fostering supportive environments that contribute to better family functioning. Fotopoulou and Parkes (19) also found that where cultural values emphasize family care, individuals using drugs receive significant support from their families. Additionally, research conducted in KwaZulu Natal, South Africa, notes that some people with opioid dependence engage in minor crimes that affect their families and so when they are on OAMT and have less need for heroin, there is less need to engage in minor acquisition crime (20).

The lack of opportunities for leisure or the constructive use of free time was a concern, and many respondents mentioned boredom as a challenge. Facilitating meaningful engagement and participation in life should be prioritized to mitigate boredom (21).

Concerns about work emphasizes the challenge of sustaining livelihoods in an environment of uncertainty and extremely high unemployment rates (22). It was also noted that not many people attend skills training sessions. The reason for this low percentage is that there is a lack of consistent skills training opportunities at COSUP sites, and this should receive more attention in future. Learnerships for specific skills development in collaboration with the South African Department of Labour could be investigated, along with better understanding supportive employment.

In addition, feeling safe and having access to safe spaces are important considerations (23). This certainly reinforces the benefit of community-based harm reduction program. More work can be done to better understand safe spaces in this context.

The COSUP Tool may not be the only assessment tool developed specifically for substance use populations, but, as far as the authors are aware, it may be the first tool that used the ICF framework in the development of the client-reported questions and demographic data fields. Instruments such as the Addiction Severity Index (24) focuses on the addiction patterns, family relationships and psychiatric status with little emphasis on the environment-activities-participation dynamic. The Mental Health Quality of Life Questionnaire (25) includes questions that are applicable for people using substances (e.g., self-image, independence, mood, relationships, daily activities, physical health, and hope for the future), but there is no assessment of the bio-psycho-social interaction in a harm reduction approach, as explained above.

What is clear is that assessment of outcomes to address drug- and related harms aligns well with the ICF framework and the principles PROMs and PREMs. Although the COSUP Tool has limited questions about COSUP clients’ experiences with the service (PREMs), it nevertheless reflects their perspectives and experiences by focusing on outcomes that matter most to them. Substance use is highly individualized, and the COSUP Tool demonstrates a commitment to client-centered care, embracing a holistic approach to understanding the complex needs of people using drugs. For individuals in substance use harm reduction programs, this tool facilitates care that is more responsive and adaptable to their evolving needs, particularly in areas of psychosocial and mental health support.

The COSUP Tool is in the process of being validated, but we foresee that it could become a useful PROM tool to track changes in the functioning of clients and investigate associations between variables. For example, when addressing clients’ physiological needs when they start the program (i.e., managing withdrawal symptoms through OAMT) and providing psycho-social intervention (such as counselling and group therapy), will clients show improvement in their participation in daily activities, and will they be better prepared to engage in skills development for sustaining livelihoods? The COSUP Functioning Tool can give insight into this process, emphasizing that, apart from the essential bio-medical interventions, the psycho-social-spiritual aspects within a context are also key.

The application of the ICF within a harm-reduction program further resonates with the Community Oriented Primary Care (COPC) approach, which is the foundation of COSUP. COPC is based on five principles, which are (1) local health and institutional analysis; (2) comprehensive care; (3) equity; (4) practice with science; and (5) person-centered care (26). This means that services must be relevant to- and in partnership with the local setting; services provided must be accessible—harm reduction programs provide an evidence-based low threshold to entry; and the program considers- and is responsive to the whole person in their context. The ICF gives expression to COPC in that it helps with understanding the interconnectedness between substance use, socio-economic factors, and health outcomes in relation to the person and the community (27).

The ICF is widely applied in clinical interprofessional practice and in tool development. In-depth training through the ICF Facilitator's course (28), developed by the WHO Collaborating Centre for the Family of International Classifications (WHO-FIC) at the National Institute for Health and the Environment (RIVM) in The Netherlands, and presented by the WHO-FIC Collaborating Center in South Africa, was instrumental in understanding the ICF as a framework in relation to the functioning of clients in a harm reduction program in South Africa. This work demonstrated that the ICF framework could be applied to the development of a unique tool within a community-based harm-reduction program with marginalized groups in a resource-constrained setting.

The study has several limitations that should be addressed in future research. As noted above, there are limited questions on the experience of the practical aspects of the service and we should consider more questions such as accessibility, quality of interaction with service providers, and range of services. In addition, we should include data analysis on how respondents experience the tool itself. Missing data on some questions diminished the utility of the assessment at the individual and program level. In future, regular training of COSUP staff is essential in assisting clients to complete all the questions in the COSUP Tool. Limited budget and access to digital tools necessitated a paper-based data collection process, which required significant human resources. Yearly rollouts were impractical and delayed the integration of the Tool into the COSUP reporting system. Going forward, the COSUP Tool will be incorporated into the baseline assessment of COSUP clients and will be done every six months as part of the required assessments to be conducted by social workers with all clients at each site. It is important to provide regular training in the ICF framework for those who work in the COSUP program to ensure holistic and consistent care.

## 5 Conclusion

Developing the COSUP Client Functioning Tool by basing it on the ICF framework has provided us with a useful picture of the functioning of people who use/d drugs in their contexts−the circumstances in which they function, how they function in their day-to-day lives, what their concerns are, and how things have changed for them since joining COSUP. This is helpful so that interventions can be responsive to clients’ needs, and the benefits of the program can be articulated in a holistic way, thus contributing to the continuation of services that impact the functioning of people who use/d drugs in South Africa.

## Data Availability

The datasets presented in this article are not readily available because of the sensitive nature of substance use research. Requests to access the datasets should be directed to the corresponding author.
